# Supplementary material for: Tail assembly interference is a common strategy in bacterial antiviral defenses
Source: Nat Commun. 2024 Aug 30;15:7539. doi: 10.1038/s41467-024-51915-4 (PMC11364771; doi:10.1038/s41467-024-51915-4)
Supplement: Supplementary file 1 — Supplementary information [file 41467_2024_51915_MOESM1_ESM.pdf]

Table S1. Phages used in this study.

Phage	Life cycle	Host	Phage nomenclature (Virus; Realm; Kingdom; Phylum; Class; Genus; Species)	Identifier	Family (previous classification)
T4	Lytic	*E. coli*	Viruses; Duplodnaviria; Heunggongvirae; Uroviricota; Caudoviricetes; Straboviridae; Tevenvirinae; Tequatrovirus; Tequatrovirus T4	AF158101	*Myoviridae*
T5	Lytic	*E. coli*	Viruses; Duplodnaviria; Heunggongvirae; Uroviricota; Caudoviricetes; Demerecviridae; Markadamsvirinae; Tequintavirus; Tequintavirus T5	AY543070	*Demerecviridae*
T7	Lytic	*E. coli*	Viruses; Duplodnaviria; Heunggongvirae; Uroviricota; Caudoviricetes; Autographiviridae; Studiervirinae; Teseptimavirus; Teseptimavirus T7	V01146	*Autographiviridae*
HK578	Temperate	*E. coli*	Viruses; Duplodnaviria; Heunggongvirae; Uroviricota; Caudoviricetes; Dhillonvirus; Dhillonvirus HK578	NC_019724.1	*Siphoviridae*
P27	Temperate	*E. coli*	Viruses; Duplodnaviria; Heunggongvirae; Uroviricota; Caudoviricetes; unclassified Caudoviricetes	AJ298298.1	*Myoviridae*
Lambda	Temperate	*E. coli*	Viruses; Duplodnaviria; Heunggongvirae; Uroviricota; Caudoviricetes; Lambdavirus; Lambdavirus lambda	NC_001416	*Siphoviridae*
phi80	Temperate	*E. coli*	Viruses; Duplodnaviria; Heunggongvirae; Uroviricota; Caudoviricetes; unclassified Caudoviricetes	JX871397.1	*Siphoviridae*
HK97	Temperate	*E. coli*	Viruses; Duplodnaviria; Heunggongvirae; Uroviricota; Caudoviricetes; Hendrixvirinae; Byrnievirus; Byrnievirus HK97	NC_002167.1	*Siphoviridae*

HK106	Temperate	*E. coli*	Viruses; Duplodnaviria; Heunggongvirae; Uroviricota; Caudoviricetes; Hendrixvirinae; Wanchaivirus; Wanchaivirus HK106	NC_019768.1	*Siphoviridae*
HK140	Temperate	*E. coli*	Viruses; Duplodnaviria; Heunggongvirae; Uroviricota; Caudoviricetes; Hendrixvirinae; Yautsimvirus; Yautsimvirus HK140	NC_019710	*Siphoviridae*
HK544	Temperate	*E. coli*	Viruses; Duplodnaviria; Heunggongvirae; Uroviricota; Caudoviricetes; Hendrixvirinae; Kwaitsingvirus; Kwaitsingvirus HK544	NC_019767.1	*Siphoviridae*
HK620	Temperate	*E. coli*	Viruses; Duplodnaviria; Heunggongvirae; Uroviricota; Caudoviricetes; Lederbergvirus; Lederbergvirus HK620	NC_002730	*Podoviridae*
P22	Temperate	*S. enterica*	Viruses; Duplodnaviria; Heunggongvirae; Uroviricota; Caudoviricetes; Lederbergvirus; Lederbergvirus P22	NC_002371.2	*Podoviridae*
BTP1	Temperate	*S. enterica*	Viruses; Duplodnaviria; Heunggongvirae; Uroviricota; Caudoviricetes; Lederbergvirus	GCA_900156925.1	*Podoviridae*
ES18	Temperate	*S. enterica*	Viruses; Duplodnaviria; Heunggongvirae; Uroviricota; Caudoviricetes; unclassified Caudoviricetes	NC_006949	

Table S2. Multiple protein sequence comparison of Tai immune systems.

	TaiEcA	TaiEcB	TaiEcC	TaiEcD	TaiSalA	TaiSalB	TaiSalC	TaiSalD	TaiSalE	TaiSalF	TaiPectA	TaiPectB
TaiEcA	100	28.32	26.84	26.11	22.78	74.02	20.29	36.46	27.47	48.76	29.41	29.95
TaiEcB		100	26.16	33.53	56.9	30.06	39.57	27.49	25.61	32.16	24.86	26.01
TaiEcC			100	21.79	30.73	27.37	26.81	24.06	25.28	31.75	26.46	26.98
TaiEcD				100	34.27	28.89	31.16	23.6	22.49	24.72	25.00	25.97
TaiSalA					100	28.33	62.59	25.28	26.32	26.4	29.41	28.88
TaiSalB						100	23.91	39.38	27.47	51.74	25.56	28.33
TaiSalC							100	21.9	22.14	23.91	24.64	21.74
TaiSalD								100	25.57	43.39	28.65	25.95
TaiSalE									100	30.77	25.00	22.80
TaiSalF										100	30.65	28.49
TaiPectA											100	63.49
TaiPectB												100

Table S3. Tai immune systems in mobile genetic elements: Genomes and characteristics (underlined systems were tested).

Phyla	Strain	Accession number (Genomic location)	Mobile Genetic Element	Localization	Tai immune version
Gammaproteobacteria	Enterobacteria phage phi80	JX871397 (44,365-44,979)	Phage	Before *ter*S	TaiEcA
Gammaproteobacteria	*E. coli* 9.1649	NZ_AEZY0200006 (51,336-51,860)	Phage	Before *ter*S	TaiEcB
Gammaproteobacteria	*E. coli* strain EK3.44 contig00006	WCBF01000006.1 (181,131-181,712)	Phage	Before *ter*S	TaiEcC
Gammaproteobacteria	*E. coli* KD1 contig00148	AJWO01000121 (92,093-92,641)	Phage	Before *ter*S	TaiEcD
Gammaproteobacteria	*Pectobacterium carotovorum* strain 67	CP034211 (1,465,892-1,466,461)	PICI-like element	After *pri*	TaiPectA
Gammaproteobacteria	*Pectobacterium polaris* strain NIBIO1006	CP017481 (4,449,297-4,449,935)	PICI-like element	After *pri*	TaiPectB
Gammaproteobacteria	*Salmonella* phage FSL SP-016	KC139516 (36,278-36,823)	Phage	Before *ter*S	TaiSalA
Gammaproteobacteria	*Salmonella enterica subsp. enterica serovar Poona strain PNUSAS026990 SAMN07982323-rid5785503.denovo.01*	AAMWLX010000001.1 (103,339-103,941)	Phage	Before *ter*S	TaiSalB
Gammaproteobacteria	*Salmonella enterica subsp. enterica serovar Bareilly* str. CFSAN000220 contig0174	JRLS01000175 (195-614)	Phage	Before *ter*S	TaiSalC
Gammaproteobacteria	*Salmonella enterica subsp. enterica serovar Newport strain CFSAN024600 SAMN03464535-rid5113533.denovo.061*	AAJQMU010000061.1 (14,435-15,016)	Phage	Between tail genes	TaiSalD
Gammaproteobacteria	*Salmonella enterica subsp. enterica serovar Ramatgan strain 153182 SAMN11109106-rid7344233.denovo.042.II*	AAIWHK010000042.1 (24,792-25,361)	Phage	Between lysis genes	TaiSalE
Gammaproteobacteria	*Salmonella enterica subsp. arizonae strain 554401 SAMN09531131-rid4897403.denovo.05*	AAIYMZ010000005.1 (24,814-25,419)	Phage	Arc-mnt region	TaiSalF
Gammaproteobacteria	*Salmonella enterica strain PNUSAS188371 SAMN17267884-rid11324653.denovo.02*	AAYRLH010000002.1 (264,164-264,733)	Phage	Between tail genes	TaiSalG
Gammaproteobacteria	*Salmonella enterica strain FLUFL-367 SAMN03102375-rid6482393.denovo.041*	AAACVH010000041.1 (21,507-22,025)	Phage	Between lysis genes	TaiSalH
Gammaproteobacteria	*Salmonella enterica serovar Waycross* strain SA20041608	CP022138 (4,123,821-4,124,660)	P4-like element	After *cos*	TaiSalI

Phyla	Strain	Accession number	Mobile Genetic Element	Localization	Tai immune version tested
Gammaproteobacteria	*Yersinia frederiksenii genome assembly 4821_8#8, scaffold ERS008455SCcontig000027*	CPZP01000027 (14,177- 14,782)	Phage	Arc-mnt region	TaiYerA
Gammaproteobacteria	*Yersinia enterocolitica genome assembly 4976_2#8, scaffold ERS008611SCcontig000003*	CTIU01000003 (35,677- 36,225)	Phage	Before *ter*S	TaiYerB
Gammaproteobacteria	*Klebsiella pneumoniae strain EuSCAPE_TR274 genome assembly, contig: 19646_395SCcontig000011*	UJYD01000011 (14,780- 15,352)	Phage	Before *ter*S	TaiKlebA
Gammaproteobacteria	*Klebsiella pneumoniae strain MJR8396D Klebsiella_spHMPREF3197-1.0_Cont353.1*	LRQC01000105 (520-1,029)	Phage	Between lysis genes	TaiKlebB
Gammaproteobacteria	*Klebsiella aerogenes strain NCTC10006*	LR134123 (1,257,367-1,257,891)	Phage	End of the phage	TaiKlebC
Gammaproteobacteria	*Klebsiella variicola strain WUSM_KV_18 NODE_8_length_279697*	QIYE01000008 (201,568-202,095)	Phage	End of the phage	TaiKlebD
Gammaproteobacteria	*Serratia marcescens strain 2880STDY5682984*	FCLJ01000001 (1,776,914-1,777,459)	Phage	Before *ter*S	TaiSerrA
Gammaproteobacteria	*Serratia fonticola strain AeS1*	MDJO01000001 (374,321-374,827)	Phage	Between tail genes	TaiSerrB
Gammaproteobacteria	*Serratia proteamaculans strain B-41162 NRRL_B41162_GTCAGTCA*	MQMT01000006 (339,139-339,648)	Phage	Before *ter*S	TaiSerrC
Gammaproteobacteria	*Serratia marcescens strain 2880STDY5683034*	FCGK01000014 (18,998-19,621)	Phage	After *ter*S	TaiSerrD
Gammaproteobacteria	*Pseudomonas putida GB-1*	CP000926 (3,838,925-3,839,500)	Phage	Arc-mnt region	TaiPseudoA
Gammaproteobacteria	*Pseudomonas sp. NFR09*	FOIH01000003 (373,395-374,009)	Phage	After int	TaiPseudoB
Gammaproteobacteria	*Pseudomonas sp. FSL R10-0056*	WIWL01000238 (9,080-9,664)	Phage	End of the phage	TaiPseudoC
Gammaproteobacteria	*Enterobacter hormaechei strain F2*	CP047570 (1,569,539-1,570,129)	Phage	Before *ter*S	TaiEnterobA
Gammaproteobacteria	*Enterobacter cloacae subsp. cloacae strain CAPREX_E7*	MWMD01000003 (732,945-733,490)	Phage	Before *ter*S	TaiEnterobB
Gammaproteobacteria	*Enterobacter hormaechei strain WCHEH090036 26*	RXRR01000026 (23,052-23,606)	Phage	Before *ter*S	TaiEnterobC
Gammaproteobacteria	*Enterobacter cancerogenus strain MiY-F*	CP045769 (1,549,525-1,550,049)	Phage	Before *ter*S	TaiEnterobD
Gammaproteobacteria	*Enterobacter cloacae strain e1272*	FJXV01000004 (377,710-378,294)	Phage	Before *ter*S	TaiEnterobE
Gammaproteobacteria	*Enterobacter cloacae strain e1343*	FJYJ01000004 (430,433-430,975)	Phage	Before *ter*S	TaiEnterobF

Phyla	Strain	Accession number	Mobile Genetic Element	Localization	Tai immune version tested
Gammaproteobacteria	*Enterobacter sp. E76*	CP042499 (3,397,117-3,397,707)	Phage	Before *ter*S	TaiEnterobG
Beta Proteobacteria	*Eikenella sp. HMSC061C02 Eikenella_spHMPREF2541-1.0_Cont230*	LTKM01000042 (34,735-35,280)	Phage	Between tail genes	TaiEikenA
Beta Proteobacteria	*Eikenella corrodens strain NML04-0072 Eikcor_contig000030*	LXSG01000030 (1,502-2,062)	Phage	Between tail genes	TaiEikenB
Beta Proteobacteria	*Eikenella sp. NML02-A-017 Eiksp_contig000032*	LXSL01000032 (161,701-162,279)	Phage	Between tail genes	TaiEikenC
Gammaproteobacteria	*Ewingella americana strain BRK18a*	LXYV01000008 (13,641-14,228)	Phage	Between tail genes	TaiEwingA
Gammaproteobacteria	*Citrobacter freundii strain GED7749C Citrobacter_spHMPREF3212-1.0_Cont1.1*	LRPR01000002 (6,937-7,533)	Phage	Before *ter*S	TaiCitroA
Gammaproteobacteria	*Citrobacter freundii strain NCTC8782*	UIGT01000001 (3,685,208-3,685,756)	Phage	Before *ter*S	TaiCitroB
Gammaproteobacteria	*Citrobacter amalonaticus strain ca_0067*	RCYA01000001 (368,052-368,633)	Phage	Before *ter*S	TaiCitroC
Gammaproteobacteria	*Citrobacter freundii complex sp. CFNIH5*	PQLH01000023 (82,457-83,032)	Phage	Between tail genes	TaiCitroD
Gammaproteobacteria	*Citrobacter sp. CFNIH10*	CP026216 (4,266,716-4,267,261)	Phage	Before *ter*S	TaiCitroE
Gammaproteobacteria	*Citrobacter freundii strain 116E5*	RZVG01000125 (35-598)	Phage	Before *ter*S	TaiCitroF
Gammaproteobacteria	*Pantoea sp. VH_24*	VWUC01000001 (214,634-215,257)	Phage	Between tail genes	TaiPantoA
Gammaproteobacteria	*Pantoea ananatis strain PNA 14-1*	QEKS01000005 (264,664-265,269)	Phage	Before *ter*S	TaiPantoB
Gammaproteobacteria	*Pantoea agglomerans strain VRA_MhP_f*	WKLC01000386 (2,525-3,076)	Phage	Before *ter*S	TaiPantoC
Gammaproteobacteria	*Pantoea ananatis strain PNA 15-1*	NMZZ01000001 (750,527-751,171)	Phage	Before *ter*S	TaiPantoD
Gammaproteobacteria	*Pantoea agglomerans strain R5*	WSST01000040 (581,782-582,426)	Phage	Before *ter*S	TaiPantoE
Gammaproteobacteria	*Pantoea ananatis DAR 76143 strain DAR76143*	NZ_BATH01000009.1 (197,967-198,524)	Phage	Before *ter*S	TaiPantoF
Gammaproteobacteria	*Hafnia alvei strain PCM_1221 856*	NZ_SISX01000071.1 (109,509-110,063)	Phage	Before *ter*S	TaiHafniA
Gammaproteobacteria	*Obesumbacterium proteus strain PCM_1214 1098*	SITL01000075 (2,771-3,325)	Phage	Before *ter*S	TaiObesuA
Gammaproteobacteria	*Kluyvera cryocrescens strain L2 contig21*	NZ_LGHZ01000021.1 (104,284-104,829)	Phage	Before *ter*S	TaiKluyvA
Gammaproteobacteria	*Lelliottia amnigena strain NCTC12124*	LR134135 (2,264,736-2,265,212)	Phage	Before *ter*S	TaiLellA

Phyla	Strain	Accession number	Mobile Genetic Element	Localization	Tai immune version tested
Gammaproteobacteria	*Cronobacter dublinensis strain cro914C1*	NROF01000006 (97,993-98,565)	Phage	Before *ter*S	TaiCronoA
Gammaproteobacteria	*Cronobacter sakazakii strain cro1539A1-2*	NRMH01000025 (11,570-12,118)	Phage	Before *ter*S	TaiCronoB

Table S4. Statistical values and quantification of particles in Figure 4a

Figure 3c	*p* value
HK544 infection MOI 0.1 594-594 phi80	< 0.0001
HK544 infection MOI 0.1 594-594 phi80 Δ*taiEcA*	0.2212
HK544 infection MOI 1 594-594 phi80	0.0005
HK544 infection MOI 1 594-594 phi80 Δ*taiEcA*	0.0232
HK544 infection MOI 10 594-594 phi80	0.0002
HK544 infection MOI 10 594-594 phi80 Δ*taiEcA*	0.1360

Figure S2d	*p* value
HK578 infection MOI 0.1 594-594 phi80	< 0.0001
HK578 infection MOI 0.1 594-594 phi80 Δ*taiEcA*	0.3226
HK578 infection MOI 1 594-594 phi80	< 0.0001
HK578 infection MOI 1 594-594 phi80 Δ*taiEcA*	0.9530
HK578 infection MOI 10 594-594 phi80	< 0.0001
HK578 infection MOI 10 594-594 phi80 Δ*taiEcA*	0.8875

Figure S4b	*p* value
Plasmid	0.4242
Phage HK106	< 0.0001

Figure S4c	*p* value
HK106 empty-TaiEcA	< 0.0001
HK106 empty-TaiEcB	< 0.0001
HK106 empty-TaiEcD	< 0.0001
HK544 empty-TaiEcA	< 0.0001
HK544 empty-TaiEcB	< 0.0001
HK544 empty-TaiEcD	< 0.0001

Figure S4d	*p* value
P27 empty-TaiEcC	0.0026
P22 empty-TaiEcC	< 0.0001

	Quantification of particles in Figure 4a			
	**HK106**		**HK544**	
	capsids	tails	capsids	tails
**pBAD18-empty**	75	81	55	54
**pBAD18-TaiEcA**	16	1	46	-
**pBAD18-TaiEcB**	22	-	36	-
**pBAD18-TaiEcC**	14	16	40	41
**pBAD18-TaiEcD**	17	-	38	-
**pBAD18-TaiPectA**	NA	NA	18	2
**pBAD18-TaiPectA**	NA	NA	33	1

Table S5. Multiple protein sequence comparison of anti-Tai immune systems.

	Anti-TaiEcA	Anti-TaiSalB	Anti-TaiYerA	Anti-TaiEnterobactA	Anti-TaiEnterobactG
Anti-TaiEcA	100	71.05	35.48	72.37	52.63
Anti-TaiSalB		100	35.48	90.79	50.00
Anti-TaiYerA			100	37.10	35.38
Anti-TaiEnterobactA				100	51.32
Anti-TaiEnterobactG					100

Table S6. Strains used in this study.

Strain	Description	Reference
594	*E. coli* laboratory strain	
DC10B	*E. coli* laboratory strain	
BTH101	*E. coli* laboratory strain	Euromedex
MG1655	*E. coli* laboratory strain	
HTD	*E. coli* laboratory strain	
JP18938	*Salmonella enterica* subsp. *enterica* serovar Typhimurium LT2 ΔFels-1 ΔGifsy-2 ΔGifsy-1 ΔFels-2	[1]
JP21561	Lysate phage T4	Gift from Ry Young
JP21542	Lysate phage T5	Gift from Jay Hinton
JP21690	Lysate phage HK578	
JP21543	Lysate phage T7	Gift from Jay Hinton
JP18983	JP18938 P22 lysogen	[1]
JP18984	JP18938 BTP1 lysogen	Gift from Jay Hinton
JP18985	JP18938 ES18 lysogen	[1]
JP10400	594 Lambda lysogen	This work
JP12507	594 phi80 lysogen	This work
JP12508	594 HK97 lysogen	This work
JP12509	594 HK106 lysogen	This work
JP12510	594 HK140 lysogen	This work
JP12511	594 HK544 lysogen	This work
JP21489	HTD HK620 lysogen	This work
JP10819	MG1655 P27 lysogen	This work
JP17691	DH5alpha pAF401	This work
JP19812	DH5alpha pAF402	This work
JP19823	DH5alpha pAF403	This work
JP19824	DH5alpha pAF404	This work
JP20984	DH5alpha pAF405	This work

JP21537	DH5alpha pAF406	This work
JP20992	DH5alpha pAF407	This work
JP20993	DH5alpha pAF408	This work
JP20985	DH5alpha pAF409	This work
JP21538	DH5alpha pAF410	This work
JP21539	DH5alpha pAF411	This work
JP21540	DH5alpha pAF412	This work
JP17692	DH5alpha pAF413	This work
JP22170	DH5alpha pAF414	This work
NK602	DH5alpha pAF415	This work
JP24605	DH5alpha pLH30	This work
JP24610	DH5alpha pLH31	This work
JP17558	DH5alpha pJP2786	This work
JP13131	594 pBAD18	This work
JP17714	594 pAF401	This work
JP19815	594 pAF402	This work
JP19845	594 pAF403	This work
JP19846	594 pAF404	This work
JP21009	594 pAF405	This work
JP21555	594 pAF406	This work
JP21010	594 pAF407	This work
JP21011	594 pAF408	This work
JP21012	594 pAF409	This work
JP21556	594 pAF410	This work
JP21557	594 pAF411	This work
JP21558	594 pAF412	This work
JP17715	594 pAF413	This work
JP19860	JP10819 pBAD18	This work
JP19861	JP10819 pAF401	This work
JP19862	JP10819 pAF402	This work
JP19863	JP10819 pAF403	This work
JP19864	JP10819 pAF404	This work

JP22172	JP10819 pAF405	This work
JP22173	JP10819 pAF406	This work
JP22174	JP10819 pAF407	This work
JP22175	JP10819 pAF408	This work
JP22176	JP10819 pAF409	This work
JP22177	JP10819 pAF410	This work
JP22178	JP10819 pAF411	This work
JP22179	JP10819 pAF412	This work
JP21509	HTD pBAD18	This work
JP21510	HTD pAF401	This work
JP21511	HTD pAF402	This work
JP21512	HTD pAF403	This work
JP21513	HTD pAF404	This work
JP22162	HTD pAF405	This work
JP22163	HTD pAF406	This work
JP22164	HTD pAF407	This work
JP22165	HTD pAF408	This work
JP22166	HTD pAF409	This work
JP22167	HTD pAF410	This work
JP22168	HTD pAF411	This work
JP22169	HTD pAF412	This work
JP20973	JP18938 pBAD18	This work
JP20974	JP18938 pAF401	This work
JP20975	JP18938 pAF402	This work
JP20976	JP18938 pAF403	This work
JP20977	JP18938 pAF404	This work
JP21005	JP18938 pAF405	This work
JP21551	JP18938 pAF406	This work
JP21006	JP18938 pAF407	This work
JP21007	JP18938 pAF408	This work
JP21008	JP18938 pAF409	This work
JP21552	JP18938 pAF410	This work

JP21553	JP18938 pAF411	This work
JP21554	JP18938 pAF412	This work
JP20445	594 phi80 Δ*orf63*	This work
JP20610	594 phi80 Δ*orf64*	This work
JP13178	594 phi80 Δ*orf62-orf66*	This work
JP20446	594 phi80 Δ*orf63-orf64*	This work
JP18971	JP12509 pBAD18	This work
JP18972	JP12509 pAF401	This work
JP20003	JP12509 pAF402	This work
JP20004	JP12509 pAF403	This work
JP20005	JP12509 pAF404	This work
JP18973	JP12511 pBAD18	This work
JP18974	JP12511 pAF401	This work
JP20006	JP12511 pAF402	This work
JP20007	JP12511 pAF403	This work
JP20008	JP12511 pAF404	This work
JP22204	JP12511 pAF411`	This work
JP22205	JP12511 pAF412	This work
JP17965	594 HK106 evolved against TaiEcA	This work
JP21693	594 HK106 evolved against TaiPectA	This work
JP17782	594 HK544 evolved against TaiEcA	This work
JP21694	594 HK544 evolved against TaiPectA	This work
JP21692	JP18938 ES18 evolved against TaiSalA	This work
JP21048	JP18938 P22 evolved against TaiEcC	This work
JP24585	HK620 evolved against TaiEcC	This work
JP20764	594 phi80 Δ*ati* evolved (1)	This work
JP20765	594 phi80 Δ*ati* evolved (2)	This work
JP24577	594 phi80 Δ*ati* pAF413	This work
JP20136	594 phi80 3xflag-*orf6 (N-terminal 3xflag-orf63 (C-terminal)*	This work
JP22196	594 phi80 3xflag-*orf6 (N-terminal 3xflag-orf63 (C-terminal)* pBAD18	This work

JP22197	594 phi80 3xflag-*orf6 (N-terminal 3xflag-orf63 (C-terminal)* pAF414	This work
JP17751	JP12509 pJP2786	This work
JP21014	JP18983 pBAD18	This work
JP21015	JP18983 pAF403	This work
JP19890	594 HK106 Δ*ter*L pBAD18	[2]
JP24743	594 HK106 chimeric (*orf64*)	This work

Table S7. Plasmids used in this study.

Plasmid	Description	Reference
**pWRG99**	Amp^R. Thermosensitive plasmid with Red system of lambda phage and I-*Sce*I endonuclease under control of tetracycline-inducible promoter (P_{tetA})	[3]
**pWRG717**	Amp^R, Km^R. pBluescript II SK+ derivative, *aph* resistance cassette and I-*Sce*I cleavage site.	[3]
**pCP20**	Amp^R. Thermosensitive plasmid with FLP recombinase	[4]
**pBAD18**	Amp^R. Expression vector	[5]
**pKO3Blue**	Cm^R. Thermosensitive allelic exchange vector in *E. coli*	[6]
**pET28a**	Km^R. Expression vector	Novagen
**pUT18**	Amp^R. Bacterial Adenylate Cyclase Two-hybrid System kit	Euromedex
**pKT25**	Km^R. Bacterial Adenylate Cyclase Two-hybrid System kit	Euromedex
**pUT18C-control**	Amp^R. Bacterial Adenylate Cyclase Two-hybrid System kit	Euromedex
**pKT25-control**	Km^R. Bacterial Adenylate Cyclase Two-hybrid System kit	Euromedex
**pAF401**	pBAD18 TaiEcA	This work
**pAF402**	pBAD18 TaiEcB	This work
**pAF403**	pBAD18 TaiEcC	This work
**pAF404**	pBAD18 TaiEcD	This work
**pAF405**	pBAD18 TaiSalA	This work
**pAF406**	pBAD18 TaiSalB	This work
**pAF407**	pBAD18 TaiSalD	This work
**pAF408**	pBAD18 TaiSalE	This work
**pAF409**	pBAD18 TaiSalF	This work
**pAF410**	pBAD18 TaiSalG	This work
**pAF411**	pBAD18 TaiPectA	This work
**pAF412**	pBAD18 TaiPectB	This work
**pAF413**	pBAD18 *orf64*	This work

**pAF414**	pBAD18 *Q* phi80	This work
**pAF415**	pKO3Blue *orf64*-HK106	This work
**pLH30**	pKT25 *atiSalB*	This work
**pLH31**	pUT18 *taiSalB*	This work
**pJP2786**	pET28a *cos* HK106	This work

Table S8. Oligonucleotides used in this study.

Mutagenesis	Primers	Sequence (5'-3')
**phi80 ∆*orf62-orf66***	Ecophi80-A62-1m	CGCTATGAATAATCCGTCAGTTATTCCGGCCTTCGACTTCC GCGAAATGGTGTGTAGGCTGGAGCTGCTTCG
	Ecophi80-A66-2c	GCCAGATAACACCACCGGGGAAACATTCCATCATGATGGC CGTGCGGACACATATGAATATCCTCCTTA
**phi80 ∆*orf63***	Ecophi80-A63-1m	GTTGATTGGCTGATCGCGGCATTACAGCAGGCATTCACTGA GTGCCTGTGATAATGCAACGGCGCGCCTACCTGTGACGG
	Ecophi80-A63-12c	GGACTATTACCATGAGATTGATTTTCCATCTTTATTCGCGAG AGCAGTGGGTCCATATGAATATCCTCCTTAG
**phi80 ∆*orf64***	Ecophi80-A64-1m	CCTTGTATTCATAAATCCTCCAGGTAGCTATATGCAAATTGA AACAAAAGAGAGGGTTTCCCAGTCACGAC
	Ecophi80-A64-2c	CCAGCGCATTGACCATCGGGATACTGAAGGGAGATTCCAT CATCTCTTAGATGCTTCCGGCTCGTATGTTG
	Ecophi80-64-dimer-1m	CATAAATCCTCCAGGTAGCTATATGCAAATTGAAACAAAAGA GTAATTAGTTGATCTAAGAGATGATGGAATCTCCCTTCAGTA TCCCGATGGTCAATG
	Ecophi80-64-dimer-2c	CATTGACCATCGGGATACTGAAGGGAGATTCCATCATCTCT TAGATCAACTAATTACTCTTTTGTTTCAATTTGCATATAGCTA CCTGGAGGATTTATG
**phi80 ∆*orf63-orf64***	Ecophi80-A63-1m	GTTGATTGGCTGATCGCGGCATTACAGCAGGCATTCACTGA GTGCCTGTGATAATGCAACGGCGCGCCTACCTGTGACGG
	Ecophi80-A63-13c	CAACCTGTCTTTATATCAGGATTCATTACCTGACTATTTGTG GGTAAAGTTCGGGTCCATATGAATATCCTCCTTAG
**phi80 3xflag-*orf6 (N-terminal)***	Ecophi80-A6-1m	CTTCTGATGCCGCTGACGATTTGTTAAACACCCCCGTTTAA GAGGCTATCAGGGTTTCCCAGTCACGAC
	Ecophi80-A6-2c	GGACCATACGCAGTGTGCGCCGGGTCGCTGTTACCGAGCG GCTGGTTATGTGCTTCCGGCTCGTATGTTG
	Ecophi80-orf6-3m	CTCCAAGCAGGTAGCGCATCTTCTGATGCCGCTGACGATTT GTTAAACACCCCCGTTTAAGAGGCTATC
	Ecophi80-orf6-2m	CCCCCGTTTAAGAGGCTATCATGGATTATAAAGATCACGAT GGCGATTATAAAGATCACGATATCG
	Ecophi80-orf6-1m	GATCACGATGGCGATTATAAAGATCACGATATCGATTATAAA GATGATGATGATAAAATGGCAATTACTGAAGTTTTCACAC
**phi80 3xflag-*orf63 (C-terminal)***	Ecophi80-A63-8m	CGCGGCATTACAGCAGGCATTCACTGAGTGCCTGTGATAAT GCAACAGCCCGTACAAAACGGGCTTGGATAGGGTTTCCCA GTCACGAC
	Ecophi80-A63-7c	CGCCGAGAATCAATTAACCGAAGAAGTTTCCTTAGATAGTG ACAGTGCTTCCGGCTCGTATGTTG
	Ecophi80-63-9m	CACTGAGTGCCTGTGATAATGCAACAGCCCGTACAAAACG GGCTTGGATTTATTTATCATCATCATCTTTATAATCG
	Ecophi80-63-7m	TTATTTATCATCATCATCTTTATAATCGATATCGTGATCTTTA TAATCGCCATCGTGATCTTTATAATCTCTCTTTGTTGCGTTC ATGGT
	Ecophi80-63-8m	ATCTCTCTTTGTTGCGTTCATGGTCACATGGTAAACGAATTG GCTAAATTGGTTATCCCAG

Plasmid	Primers	Sequence (5'-3')
**pBAD18**		
**pAF401**	Ecophi80-63-3cS<br>Ecophi80-63-2mH	ACGCGTCGACCAATTTGCATATAGCTACCTG<br>CCCAAGCTTGGCATTCACTGAGTGCCTGTG
**pAF402**	orf63-likeB-1mE<br>orf63-likeB-2cS	CCGGAATTCTTGTCAGTGAGCGCCATATTTTCC<br>ACGCGTCGACCTCAGTCAATGATCTCTATTGTCC
**pAF403**	orf63-likeC-1mE<br>orf63-likeC-2cS	CCGGAATTCCATAATTGTGTGTTTAAATGTTTTTC<br>ACGCGTCGACATCCCAGATTGTTATTTGAAGAC
**pAF404**	orf63-likeD-1mE<br>orf63-likeD-2cS	CCGGAATTCCGTTCAAATTTACAAGATATTAGCAAC<br>ACGCGTCGACAGAGCCTAGCTCTGTGGCGTG
**pAF405**	Salmonella-A-1mS<br>Salmonella-A-2cH	ACGCGTCGACGAGTGCTCATCTGCCCACGAG<br>CCCAAGCTTGTGAATGCCTGCTGTAAAATG
**pAF406**	Salmonella-B-1mS<br>Salmonella-B-2cH	ACGCGTCGACGTTTGATTTTTCATATAGCTACCAGG<br>CCCAAGCTTGGTCATTCAAGACTTTCTGTGC
**pAF407**	Salmonella-D-1mS<br>Salmonella-D-2cH	ACGCGTCGACGAAGGGTGATAAACATTATCGTTAG<br>CCCAAGCTTCAGATGGATTCCCGTCAATTG
**pAF408**	Salmonella-E-1mS<br>Salmonella-E-2cH	ACGCGTCGACCACCATCAATACTAACCTTGTAG<br>CCCAAGCTTTGCTGACGTGTAAAGGGATG
**pAF409**	Salmonella-F-1mS<br>Salmonella-F-2cH	ACGCGTCGACGATCACCATAGTGTCATTAATATTC<br>CCCAAGCTTGACAGGAAATAGGTTTCTGGAG
**pAF410**	Salmonella-G-1mS<br>Salmonella-G-2cH	ACGCGTCGACTCCCGTCATTTGCGTGAAGCA<br>CCCAAGCTTCCATCAATAAATTGTCTCAGTAGG
**pAF411**	PectobacteriumC-A2-1mS<br>PectobacteriumC-A2-2cH	ACGCGTCGACCTTTAAGAAGGAGATATACCATGAACATCCAGAGCTTCATC<br>CCCAAGCTTCCTGAAGTAAACAATACCGTG
**pAF412**	PectobacteriumP-A3-1mS<br>PectobacteriumP-A3-2cH	ACGCGTCGACCTTTAAGAAGGAGATATACCATGAGCCTGAATGTTCGTTGGC<br>CCCAAGCTTGGAATTGATTGCCTCGGTAGC
**pAF413**	Ecophi80-64-1mS<br>Ecophi80-64-2cH	ACGCGTCGACTCGCTTTCCACTGCTCTCGCG<br>CCCAAGCTTTATCAGGATTCATTACCTGAC
**pAF414**	Ecophi80-57-1mS<br>Ecophi80-57-2cH	ACGCGTCGACATGCCGCCAATAGTAAAGCAC<br>CCCAAGCTTCGAAATGCAATACCTATTTCC
**pKO3Blue**		
**pAF415**	HK106-orf64-1mB<br>HK106-orf64-2c<br>HK106-orf64-3m	CGCGGATCCATATGCAGAAGCGTCAACGTG<br>GGATTTATGAATACAAGGATTTTCATAGCGTCTTTACCTTTTAGAAAGTG<br>CACTTTCTAAAAGGTAAAGACGCTATGAAAATCCTTGTATTCATAAATCC

	HK106-orf64-4c	GTTATGAAAATTGCTCTAAACAAGCACTATTTGTGGGTAAAGTTCGTAGTGC
	HK106-orf64-5m	GCACTACGAACTTTACCCACAAATAGTGCTTGTTTAGAGCAATTTTCATAAC
	HK106-orf64-6cNotI	ATAAGAATGCGGCCGCCGGGGCTCTTTGTTCATGCC
**pKT25**		
**pLH30**	TaiSalBlike-64-1-1mB	CGCGGATCCCATGAAAAATCAAACAAAAGAGATGGTG
	TaiSalBlike-64-1-2cK	GGGGTACCCGTTTGCGGTTAATGTCCATCG
**pUT18**		
**pLH31**	TaiSalBlike-1mB	CGCGGATCCCATGAATGCAAGGATTTTTATGGATTAC
	TaiSalBlike-2cK	GGGGTACCCGCCCTTGTTTCACTTTAGC
**pET28a**		
**pJP2786**	HK106-siteCos-1mE	CCGGAATTCGACATTGACCGTGTCAGGAC
	HK106-siteCos-2cH	CCCAAGCTTTCGGCAGGCGTCCAGCTGTCC

**Southern blot**	**Primers**	**Sequence (5'-3')**
**Phage HK106 probe**	HK106-int-2m	CAATTTTGTCCCACTCCCTG
	HK106-int-2c	GACTTACAGCTGACGAATAC

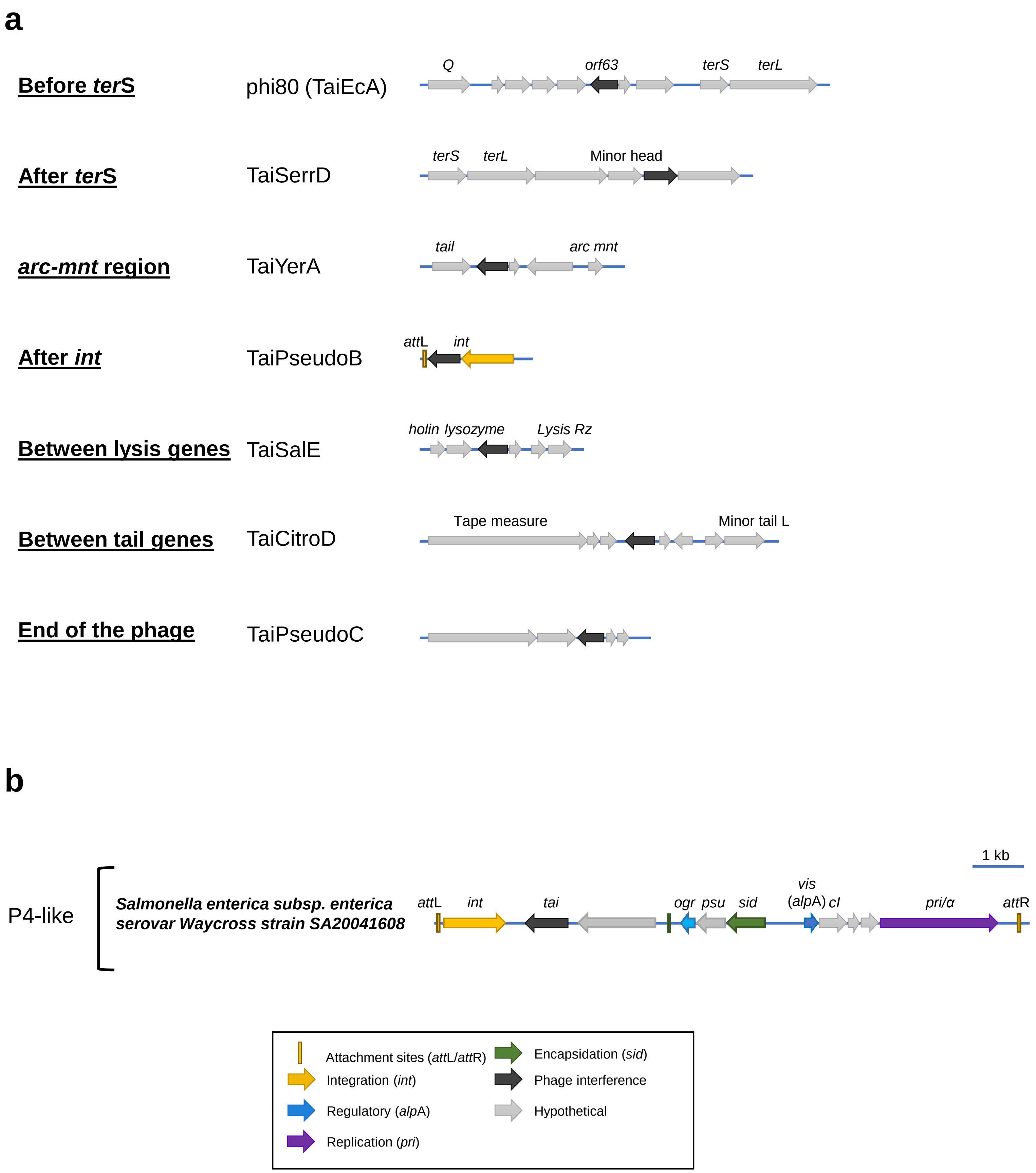

Figure S1. Phages and P4-like elements encode the Tai immune system in different hot spots of interference

Genomes are aligned according to the prophage convention, with the integrase gene (*int*) at the left end. Genes are coloured according to their sequence and function: *int* is yellow; transcription regulator is blue; replication gene is purple; encapsidation genes are green; genes encoding putative phage resistance proteins are black; genes encoding hypothetical proteins are grey. Genome maps for phages **(a)** and for P4-like elements **(b)**.

a

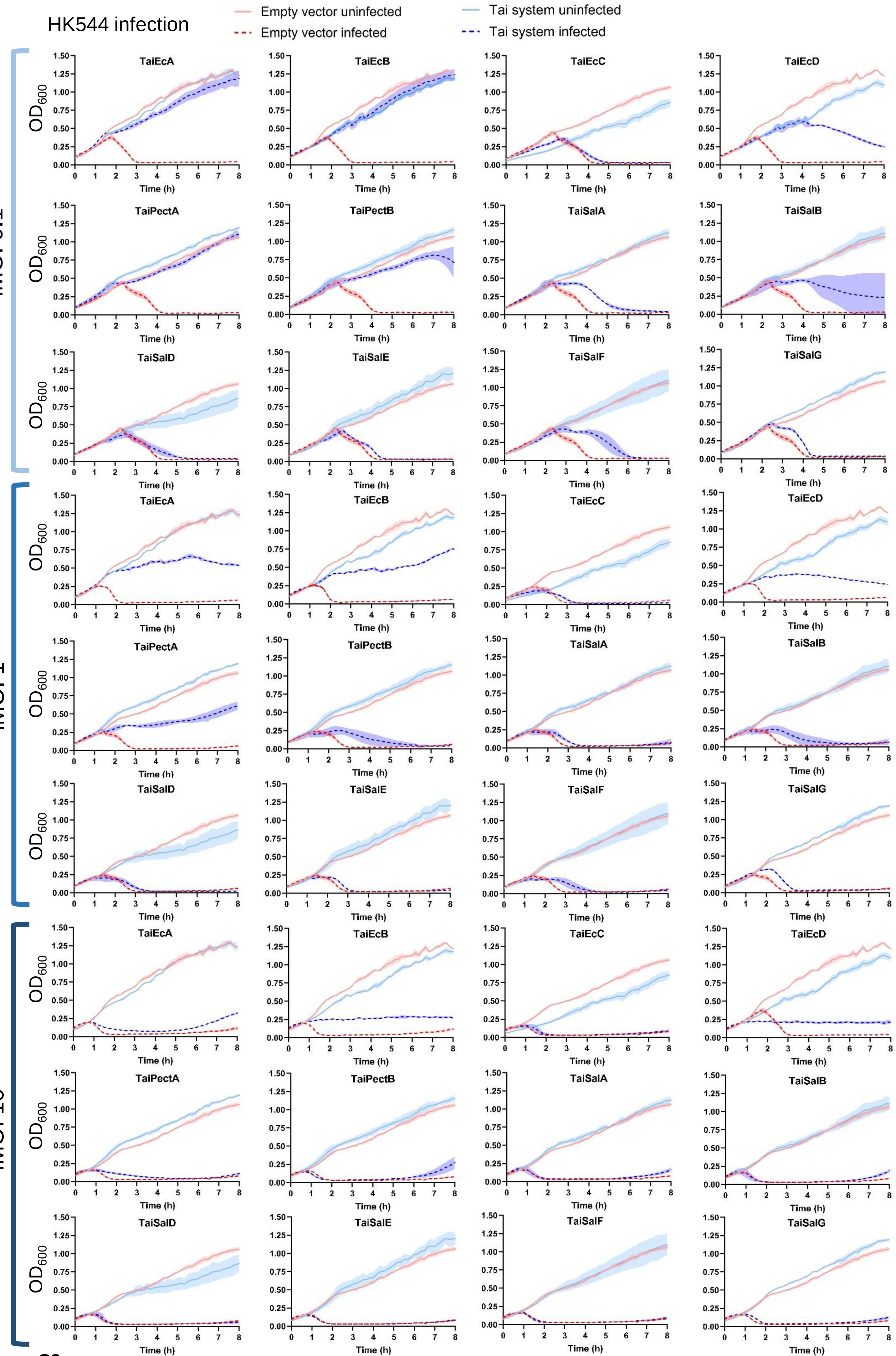
HK544 infection
Empty vector uninfected
Empty vector infected
Tai system uninfected
Tai system infected
iMOI 0.1
iMOI 1
iMOI 10
TaiEcA
TaiEcB
TaiEcC
TaiEcD
TaiPectA
TaiPectB
TaiSalA
TaiSalB
TaiSalD
TaiSalE
TaiSalF
TaiSalG
OD_{600}
Time (h)

Figure S2.

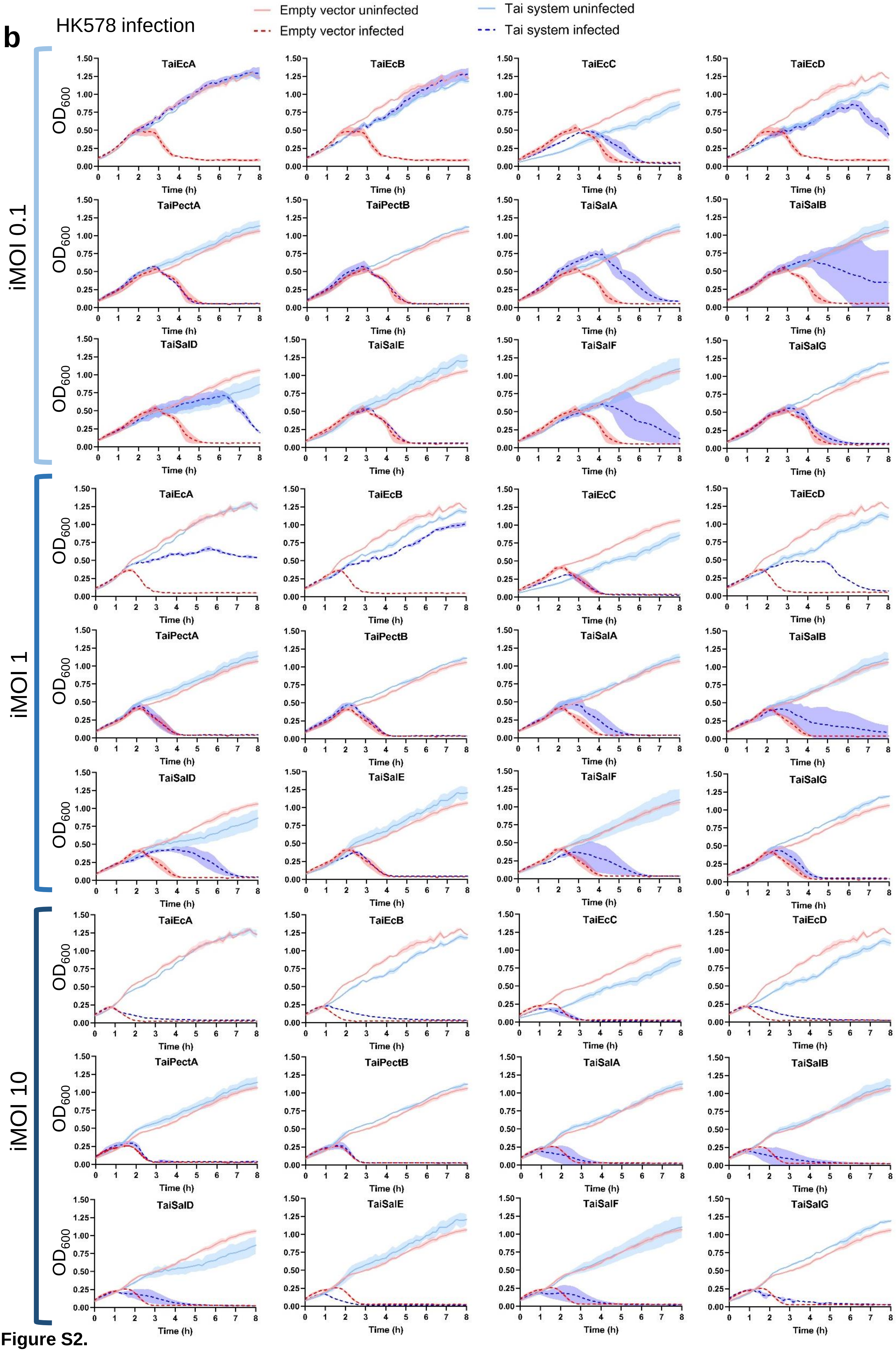
b
HK578 infection
Empty vector uninfected
Empty vector infected
Tai system uninfected
Tai system infected
iMOI 0.1
iMOI 1
iMOI 10
OD600
Time (h)
TaiEcA
TaiEcB
TaiEcC
TaiEcD
TaiPectA
TaiPectB
TaiSalA
TaiSalB
TaiSalD
TaiSalE
TaiSalF
TaiSalG

Figure S2.

c

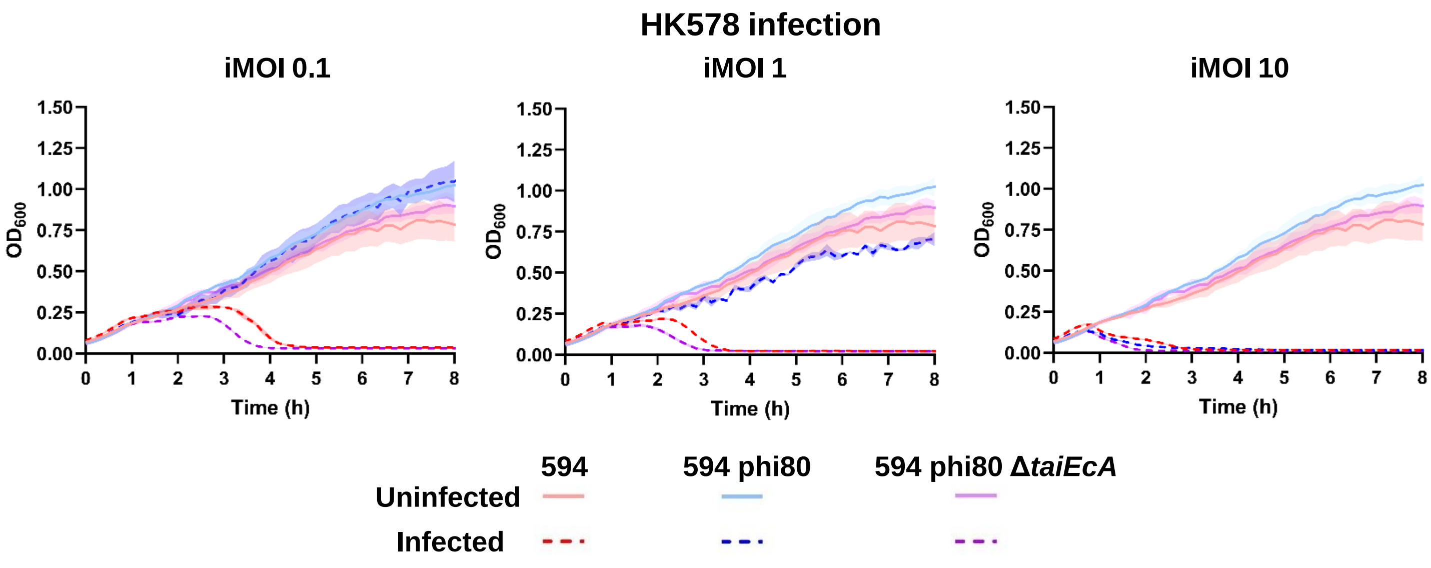

HK578 infection
iMOI 0.1
iMOI 1
iMOI 10
OD600
1.50
1.25
1.00
0.75
0.50
0.25
0.00
0 1 2 3 4 5 6 7 8
Time (h)
594
594 phi80
594 phi80 ΔtaiEcA
Uninfected
Infected

d

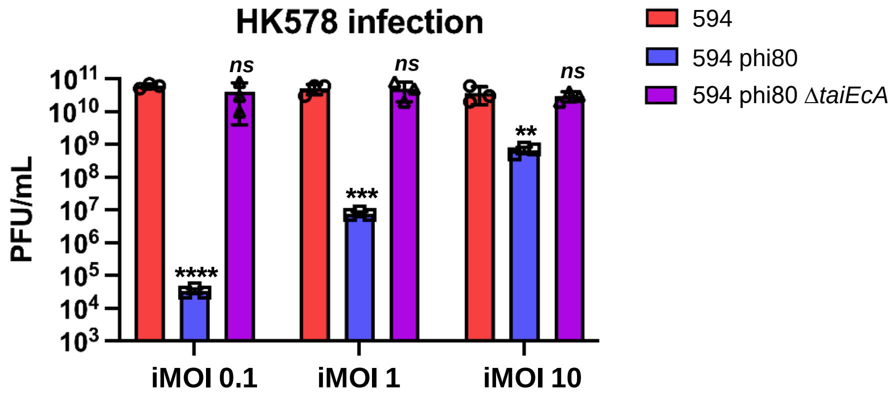

HK578 infection
594
594 phi80
594 phi80 ΔtaiEcA
PFU/mL
10^11
10^10
10^9
10^8
10^7
10^6
10^5
10^4
10^3
****
ns
***
ns
**
ns
iMOI 0.1
iMOI 1
iMOI 10

Figure S2. Growth curve infection assays with HK544 and HK578 phages

Strains with empty plasmid or encoding the different Tai immune versions were infected with HK544 **(a)** or HK578 **(b)** at an input MOI (iMOI) of 0.1, 1 or 10. **c,** Non-lysogenic or lysogenic strains for phi80 and phi80 Δ*taiEcA* was infected with HK578 phage at an iMOI of 0.1, 1 or 10. **d,** Phage titers were determined from (c) using *E. coli* 594 as recipient. For (a), (b) and (c), the means and SD of three independent experiments are represented (n = 3). For (d), the means of the phage-forming units (PFUs) and SD of three independent experiments are represented (n = 3). A one-way ANOVA with Dunnett's multiple comparisons test was performed to compare results from phage infection in a non-lysogenic strain against lysogenic strains. Adjusted *p* values were as follows: $ns > 0.05$; $*p \leq 0.05$; $**p \leq 0.01$; $***p \leq 0.001$; $****p \leq 0.0001$. The exact statistical values for each of the conditions tested are listed in Table S4. Source data are provided as a Source Data file.

a

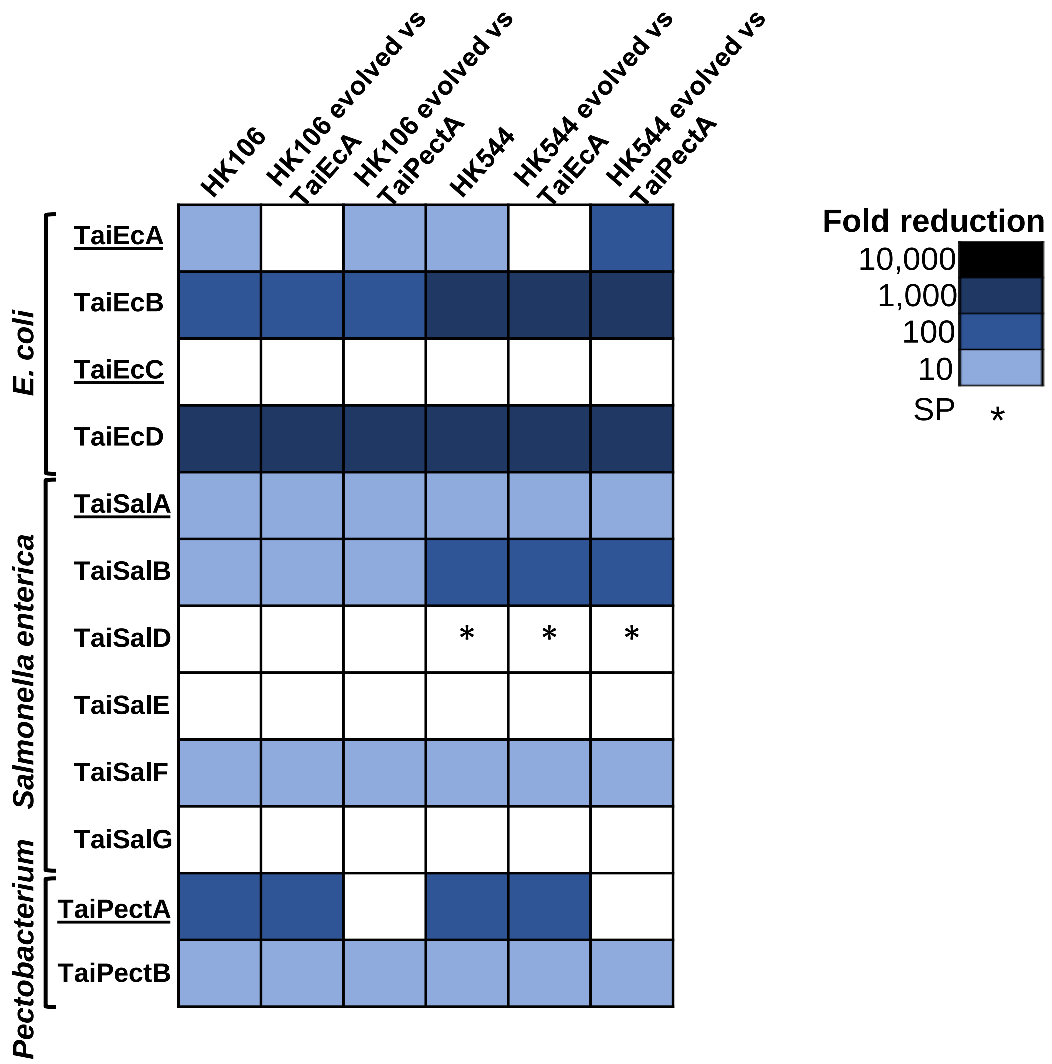

HK106
HK106 evolved vs TaiEcA
HK106 evolved vs TaiPectA
HK544
HK544 evolved vs TaiEcA
HK544 evolved vs TaiPectA
E. coli
TaiEcA
TaiEcB
TaiEcC
TaiEcD
Salmonella enterica
TaiSalA
TaiSalB
TaiSalD
TaiSalE
TaiSalF
TaiSalG
Pectobacterium
TaiPectA
TaiPectB
Fold reduction
10,000
1,000
100
10
SP
*

b

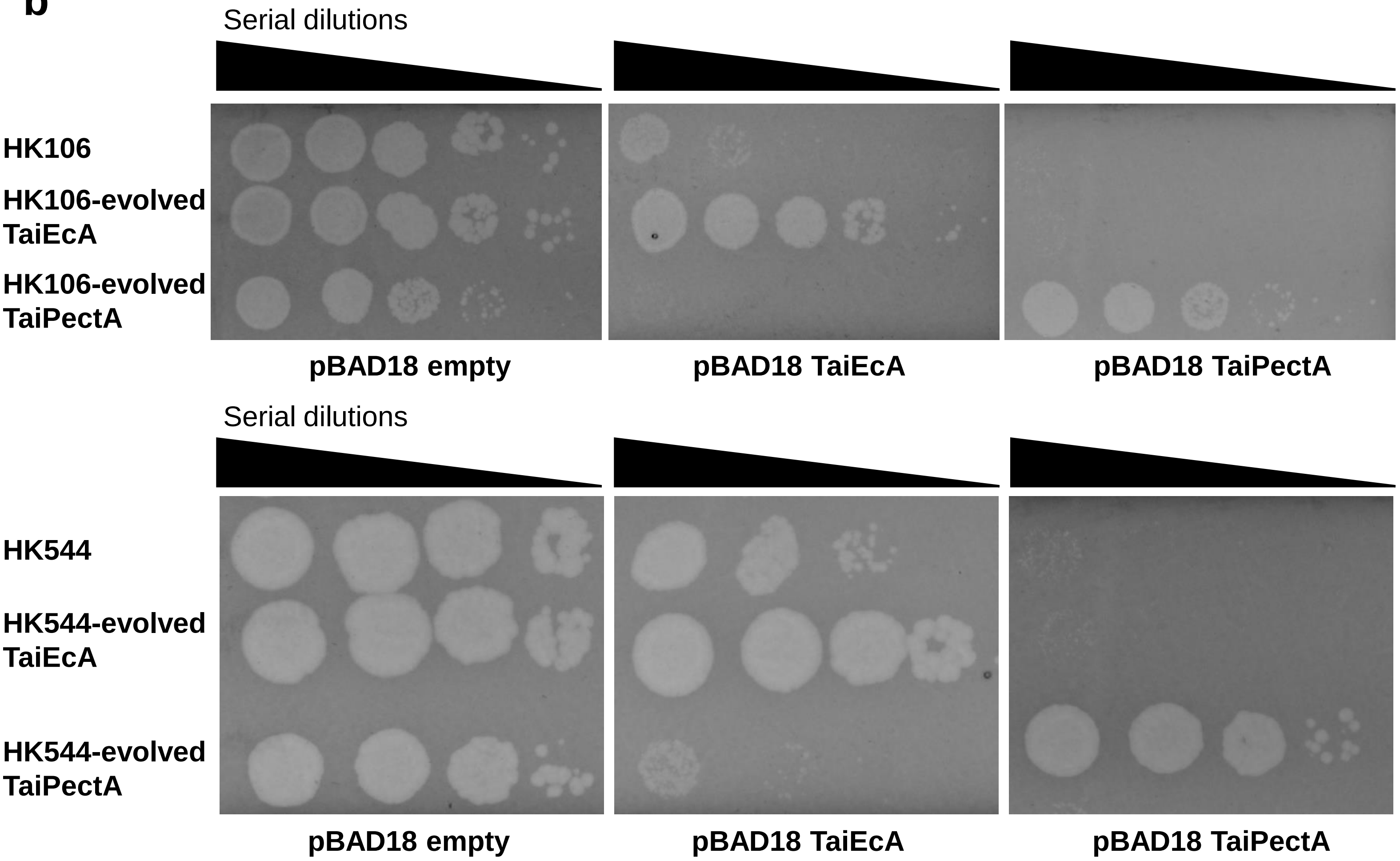

Serial dilutions
HK106
HK106-evolved TaiEcA
HK106-evolved TaiPectA
pBAD18 empty
pBAD18 TaiEcA
pBAD18 TaiPectA
Serial dilutions
HK544
HK544-evolved TaiEcA
HK544-evolved TaiPectA
pBAD18 empty
pBAD18 TaiEcA
pBAD18 TaiPectA

Figure S3.

Figure S3. Evolved phages are only insensitive against the immune system from where they were originated

a, Tai immune versions from *E. coli*, *Salmonella enterica* and *Pectobacterium* were challenged against phages HK106, HK544 and evolved phages HK106 and HK544. Heatmap represents the phage fold-change protection, which was measured using serial dilution spot assay plaque. To calculate the fold-change reduction, it was compared the efficiency of phage's plating on strains carrying either the empty plasmid or the plasmid expressing the immune system tested. The data is representative of three replicates. SP represents small plaques phenotype. **b,** Phages HK106, HK544 and evolved phages, were spotted on non-lysogenic *E. coli* 594, containing empty plasmid, pBAD18 *taiEcA* or pBAD18 *taiPectA*. Plates were supplemented with 0.02% arabinose. Ten-fold phage dilutions are shown. Experiment was repeated twice with similar results. Source data are provided as a Source Data file.

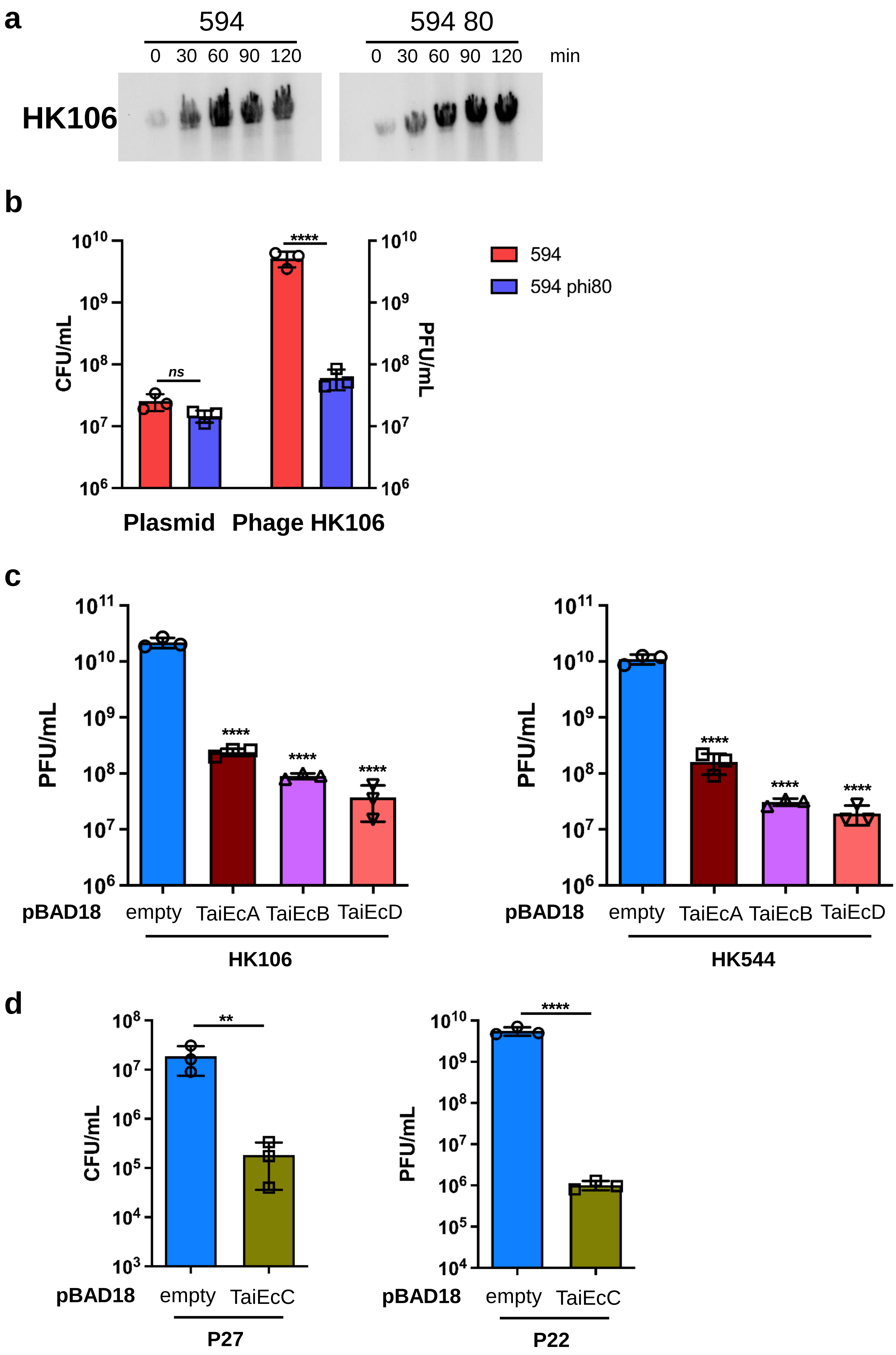
a
594
594 80
0 30 60 90 120
0 30 60 90 120
min
HK106
b
CFU/mL
PFU/mL
ns
****
594
594 phi80
Plasmid
Phage HK106
c
PFU/mL
****
pBAD18
empty
TaiEcA
TaiEcB
TaiEcD
HK106
HK544
d
CFU/mL
PFU/mL
**
****
empty
TaiEcC
P27
P22

Figure S4.

Figure S4. Tai immune systems do not act as superinfection exclusion systems

a, Non-lysogenic strain *E. coli* 594, or its derivative carrying phi80, were infected an input MOI (iMOI) of 1 with phage HK106. The DNA was extracted at different time points, resolved on a 0.7% agarose gel, and a Southern blot using phage HK106-specific probes was performed. Experiment was repeated twice with similar results. **b,** Lysogenic strain for phage HK106 carrying pJP2786 was MC (2 mg/mL) induced, and the phage and plasmid titers were determined using *E. coli* 594 or *E. coli* 594 80 as recipient strains. An unpaired two-sided *t* test was performed to compare mean differences between results in non-lysogenic and lysogenic strains. **c,** Lysogenic strains for phages HK106 or HK544, carrying different pBAD18 versions with immune systems, were MC (2 mg/mL) and arabinose (0.02%) induced, and the phage titers determined using *E. coli* 594. A one-way ANOVA with Dunnett's multiple comparisons test was performed to compare empty plasmid against different *tai* versions. **d,** Lysogenic strains for phages P27 or P22, carrying empty pBAD18 plasmid or derivatives expressing TaiEcC, were MC (2 mg/mL) and arabinose (0.02%) induced, and the phage titers were determined using *E. coli* 594 for phage P27 or *Salmonella enterica* LT2 for P22. An unpaired two-sided *t* test was performed to compare mean differences between empty plasmid and TaiEcC.

For (b), (c), and (d), the means of the phage-forming units (PFUs) or the colony-forming units (CFUs) and SD of three independent experiments are represented (n = 3). Adjusted *p* values were as follows: *ns*>0.05; *$p \leq 0.05$; **$p \leq 0.01$; ***$p \leq 0.001$; ****$p \leq 0.0001$. The exact statistical values for each of the conditions tested are listed in Table S4. Source data are provided as a Source Data file.

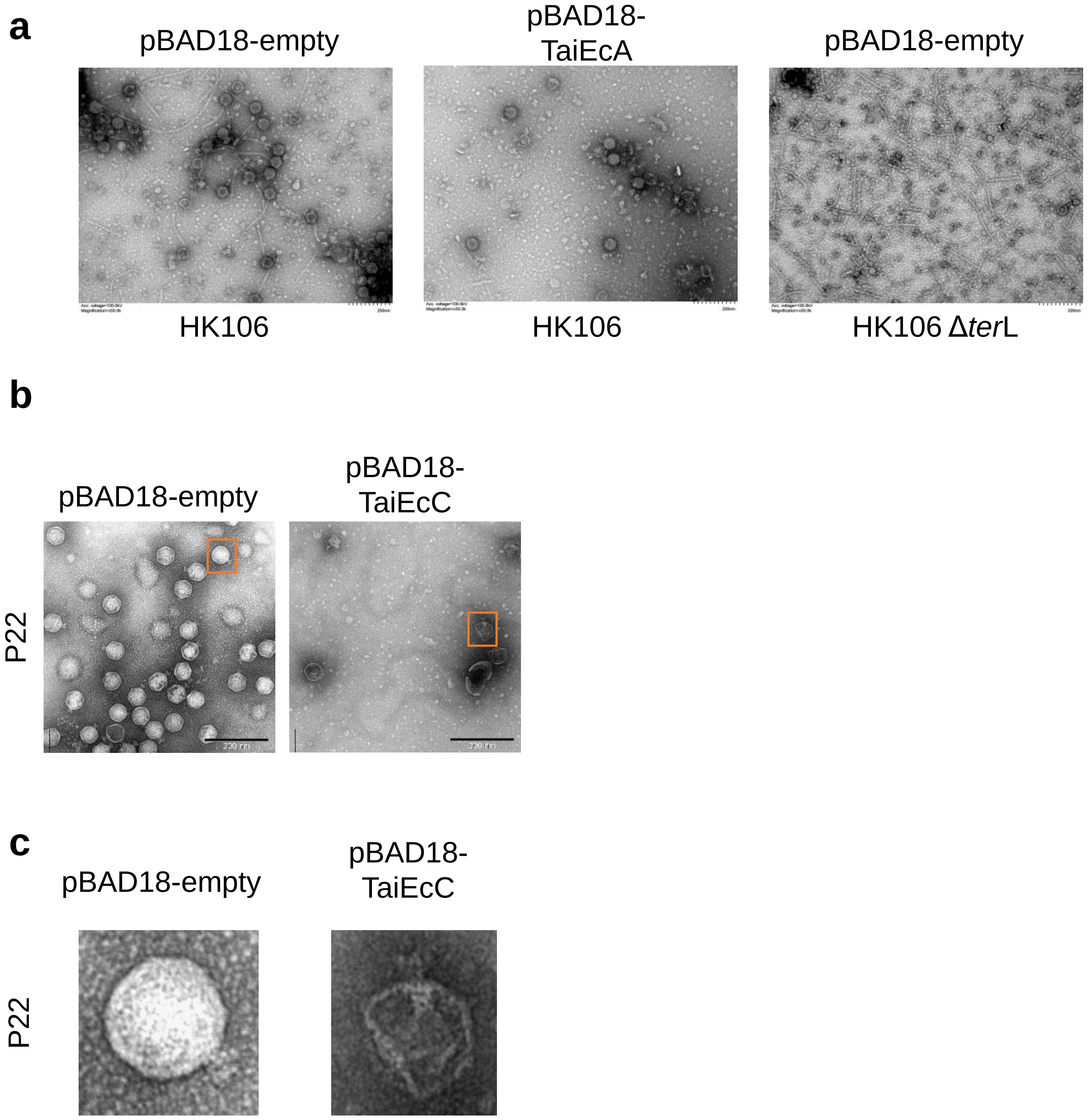

Figure S5. Tai immune system inhibits the tail assembly pathway of different phages

a, Electron microscopy images of PEG precipitated HK106 and HK106 Δ*ter*L phages obtained after MC-induction of lysogenic strains in presence of absence of TaiEcA. **b,** Electron microscopy analyses of purified phage P22 particles resulting from infection of a strain carrying or not the TaiEcC immune system. **c,** Zoom of a P22 particle highlighted in orange square from (a) is visualized, displaying a phage with or without encapsidated dsDNA. For (a), (b), and (c), different fields are shown, containing complete virion particles or tailless particles. Experiments in (a) and (b) were repeated twice with similar results. Images were collected using a Hitachi HT7800 or a JEOL 1200 TEM microscope with 12K,15K or 50K magnification. Scale bars represent 200 nm. Source data are provided as a Source Data file.

a

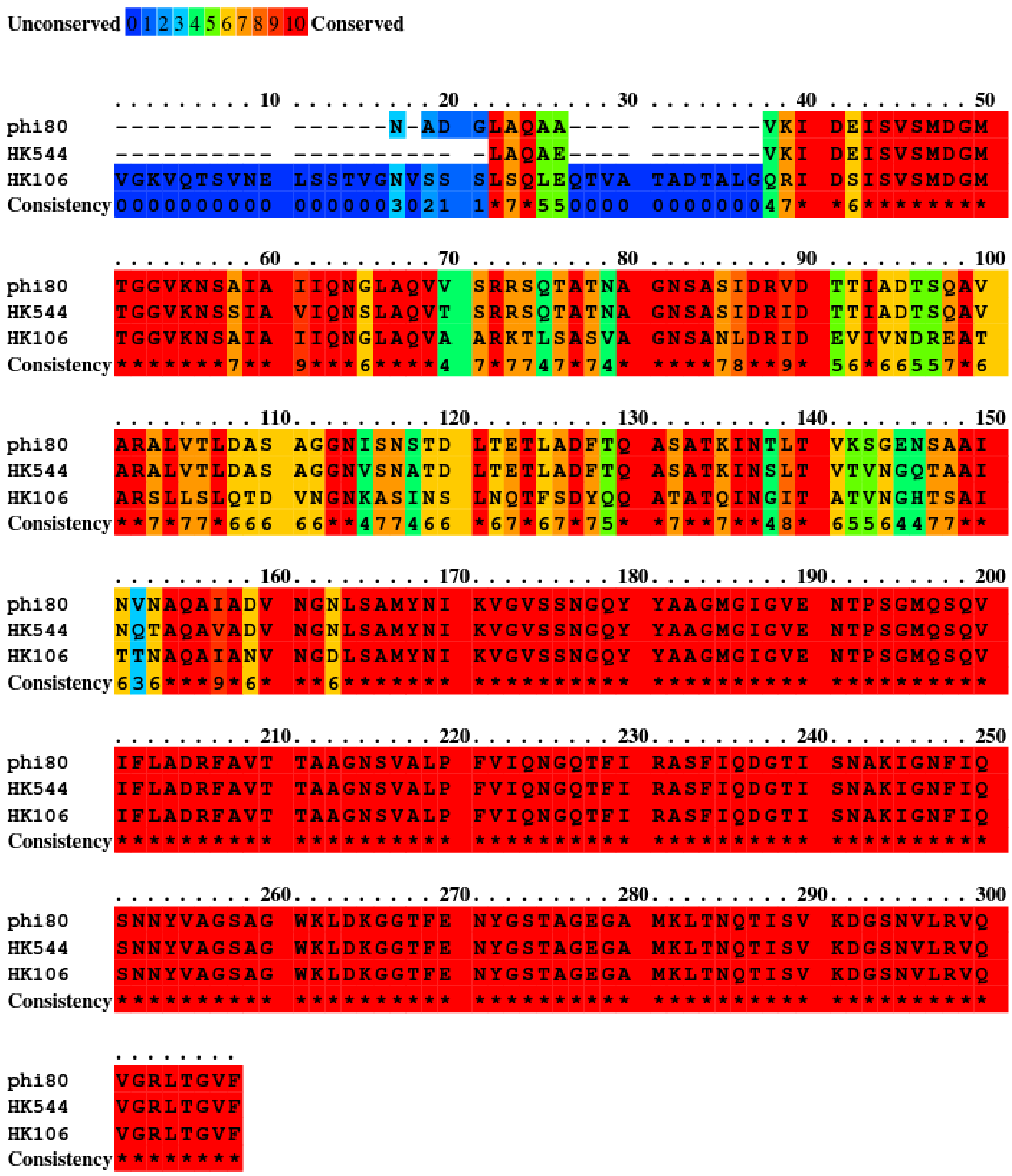

Unconserved 0 1 2 3 4 5 6 7 8 9 10 Conserved
phi80
HK544
HK106
Consistency

Figure S6.

b

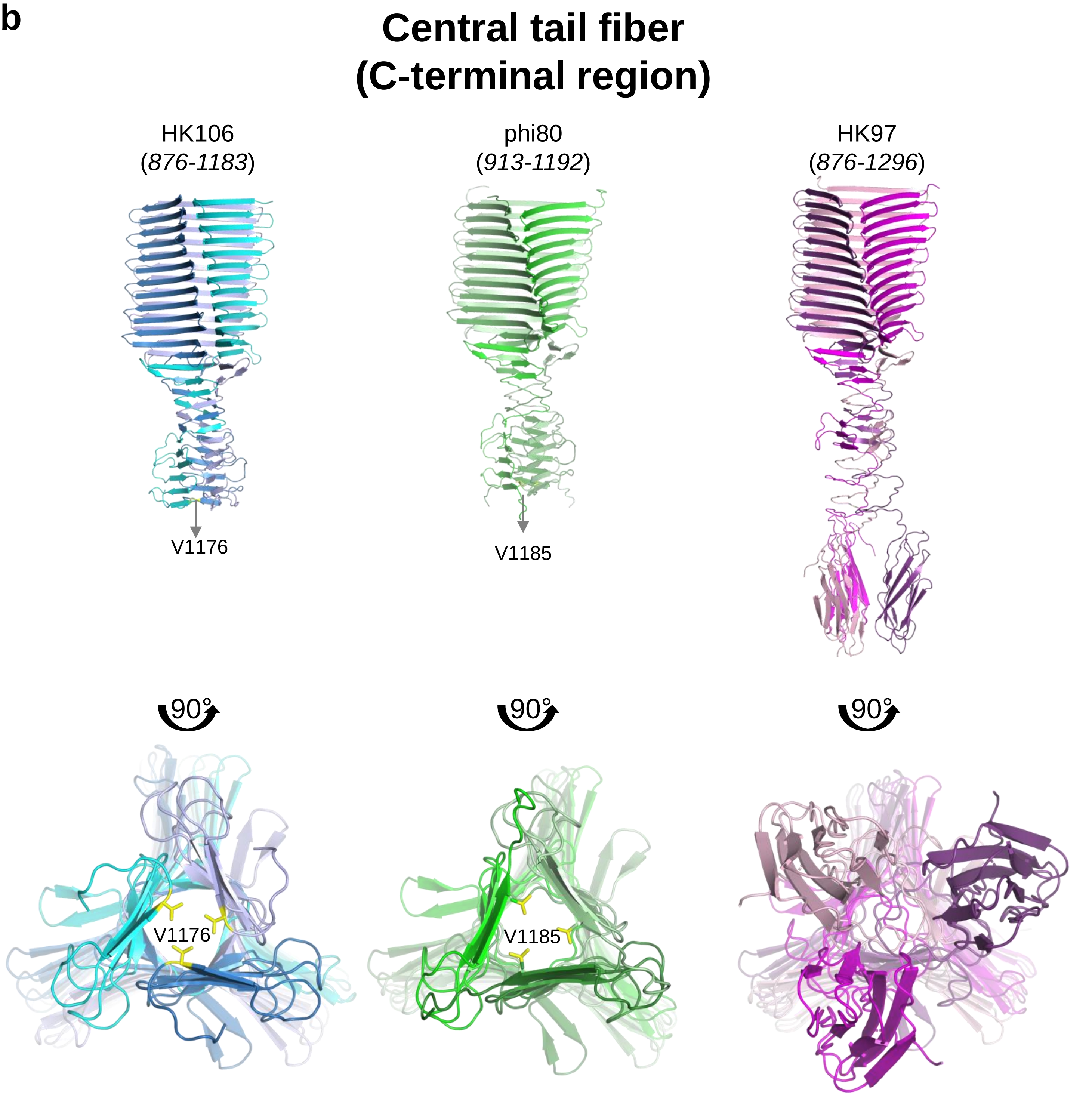

Figure S6. AlphaFold models of the central tail fiber for HK106, phi80 and HK97 phages

a, Protein sequence alignment of the C-terminal region of the J protein encoded by phages phi80, HK106 and HK544, generated using the PRALINE server. Colors indicate relative sequence conservation at each position, with red being most conserved and blue least. **b,** The predicted structure of the C-terminal region of the J protein (amino acids indicated for each phage) was predicted using AlphaFold. Different views are shown, and the central tail fiber is colored according to each phage. The mutated amino acid present in phage HK106 and the equivalent amino acid located in phi80 are indicated.

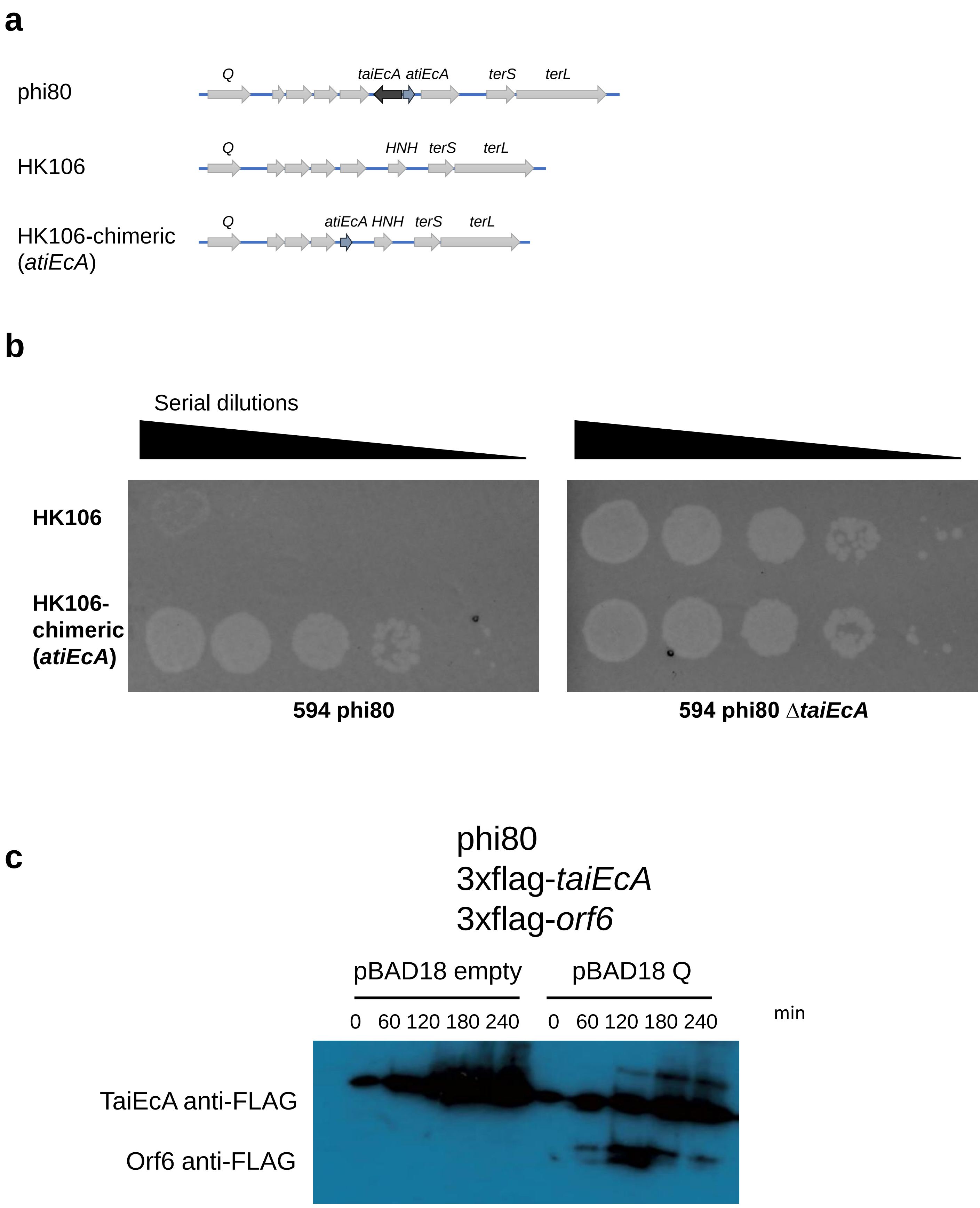

Figure S7. Counter-defense (anti-TaiEcA) avoids TaiEcA's activity

a, Schematic representation of phages phi80, HK106 and HK106-chimeric. The location of *atiEcA* (*orf64*) in each phage is highlighted. **b,** Phages HK106 and HK106-chimeric, were spotted on non-lysogenic *E. coli* 594 or *E. coli* 594 lysogenic for phi80. Ten-fold phage dilutions are shown. **c,** Lysogenic strain phi80 3xflag-*taiEcA*/3xflag-*orf6*, containing empty plasmid or pBAD18 *Q*, were arabinose induced, and samples were harvested at 0, 60, 120, 180 and 240 minutes. A western blot using antibodies against to the FLAG-tag carried by TaiEcA and Orf6 was performed. The protein molecular weight of TaiEcA is 26.1 kDa, while for Orf6 is 14.3 kDa. Experiments in (b) and (c) were repeated twice with similar results. Source data are provided as a Source Data file.

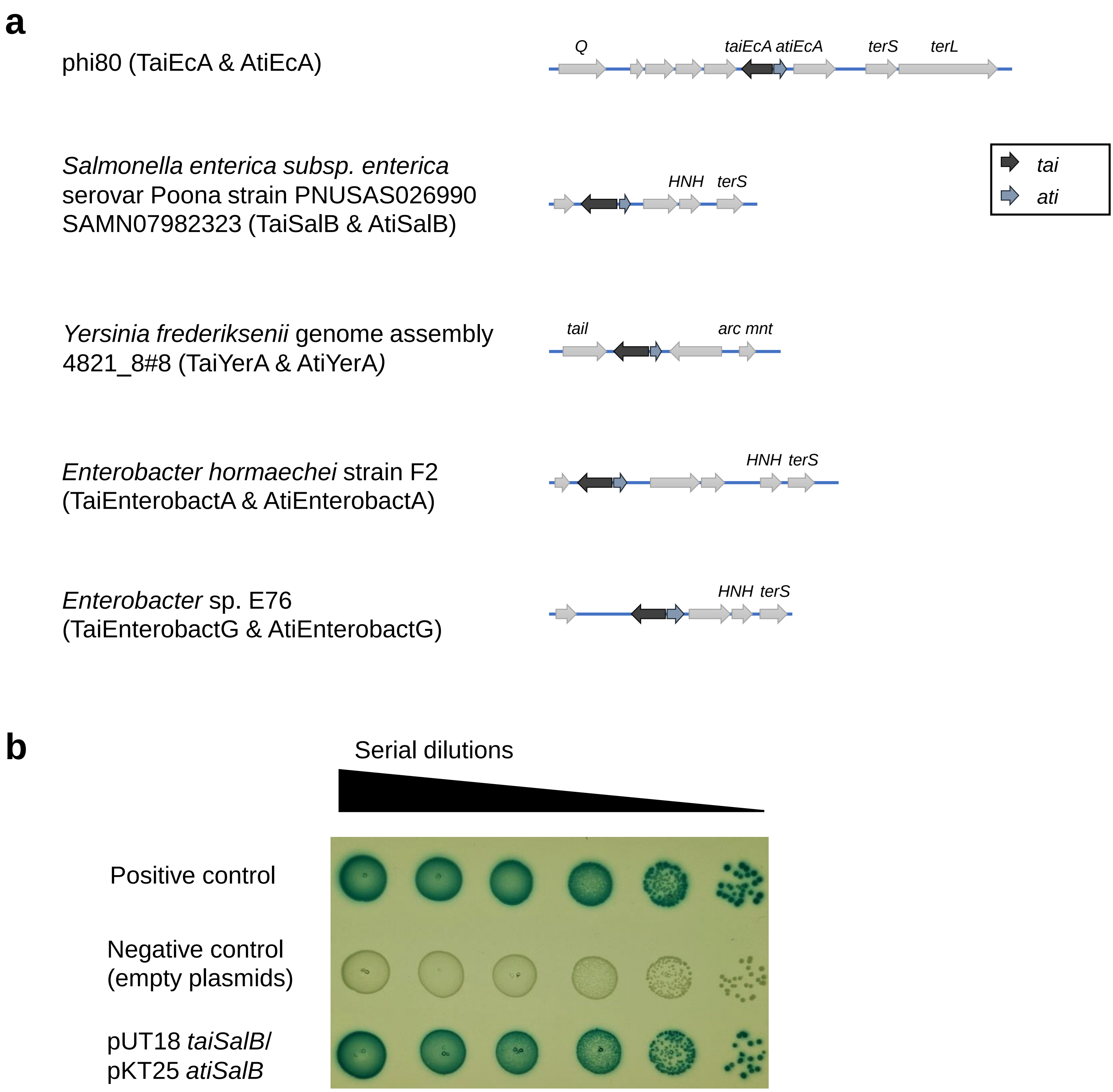

Figure S8. Multiple phages encode Tai immune system and counter-defense
a, Schematic representation of phages encoding *tai* immune system and *ati* (*orf64*-like) genes. **b,** Bacterial adenylate cyclase-based two-hybrid (BACTH) analysis was performed using the plasmid pUT18 encoding *taiSalB* and plasmid pKT25 encoding *atiSalB*. Plasmid combinations are indicated. Experiment was repeated twice with similar results. Source data are provided as a Source Data file.

Supplementary References

1. Fillol-Salom, A., Bacigalupe, R., Humphrey, S. *et al.* Lateral transduction is inherent to the life cycle of the archetypical *Salmonella* phage P22. *Nat Commun* **12**, 6510 (2021).
2. Alqurainy N, Miguel-Romero L, Moura de Sousa J, Chen J, Rocha EPC, Fillol-Salom A, Penadés JR. A widespread family of phage-inducible chromosomal islands only steals bacteriophage tails to spread in nature. *Cell Host Microbe* **31**(1):69-82.e5 (2023).
3. Hoffmann S, Schmidt C, Walter S, Bender JK, Gerlach RG. Scarless deletion of up to seven methyl-accepting chemotaxis genes with an optimized method highlights key function of CheM in Salmonella Typhimurium. *PLoS One* **12**(2):e0172630 (2017).
4. Datsenko, K. A. & Wanner, B. L. One-step inactivation of chromosomal genes in Escherichia coli K-12 using PCR products. *Proc. Natl Acad. Sci. USA* **97**, 6640-6645 (2000).
5. Guzman, L. M., Belin, D., Carson, M. J. & Beckwith, J. Tight regulation, modulation, and high-level expression by vectors containing the arabinose PBAD promoter. *J. Bacteriol.* **177,** 4121–4130 (1995).
6. Solano C, García B, Latasa C, Toledo-Arana A, Zorraquino V, Valle J, Casals J, Pedroso E, Lasa I. Genetic reductionist approach for dissecting individual roles of GGDEF proteins within the c-di-GMP signaling network in Salmonella. *Proc Natl Acad Sci USA*. **106**(19):7997-8002 (2009).
