# Supplementary material for: Tail assembly interference is a common strategy in bacterial antiviral defenses
Source: Nat Commun. 2024 Aug 30;15:7539. doi: 10.1038/s41467-024-51915-4 (PMC11364771; doi:10.1038/s41467-024-51915-4)
Supplement: Supplementary file 3 — Reporting Summary [file 41467_2024_51915_MOESM3_ESM.pdf]

nature portfolio

Corresponding author(s): José R Penadés

Last updated by author(s): Jul 19, 2024

Reporting Summary

Nature Portfolio wishes to improve the reproducibility of the work that we publish. This form provides structure for consistency and transparency in reporting. For further information on Nature Portfolio policies, see our Editorial Policies and the Editorial Policy Checklist.

Statistics

For all statistical analyses, confirm that the following items are present in the figure legend, table legend, main text, or Methods section.

n/a	Confirmed	
[ ]	[x]	The exact sample size (*n*) for each experimental group/condition, given as a discrete number and unit of measurement
[ ]	[x]	A statement on whether measurements were taken from distinct samples or whether the same sample was measured repeatedly
[ ]	[x]	The statistical test(s) used AND whether they are one- or two-sided<br>*Only common tests should be described solely by name; describe more complex techniques in the Methods section.*
[x]	[ ]	A description of all covariates tested
[ ]	[x]	A description of any assumptions or corrections, such as tests of normality and adjustment for multiple comparisons
[ ]	[x]	A full description of the statistical parameters including central tendency (e.g. means) or other basic estimates (e.g. regression coefficient) AND variation (e.g. standard deviation) or associated estimates of uncertainty (e.g. confidence intervals)
[ ]	[x]	For null hypothesis testing, the test statistic (e.g. *F*, *t*, *r*) with confidence intervals, effect sizes, degrees of freedom and *P* value noted<br>*Give P values as exact values whenever suitable.*
[x]	[ ]	For Bayesian analysis, information on the choice of priors and Markov chain Monte Carlo settings
[x]	[ ]	For hierarchical and complex designs, identification of the appropriate level for tests and full reporting of outcomes
[x]	[ ]	Estimates of effect sizes (e.g. Cohen's *d*, Pearson's *r*), indicating how they were calculated

Our web collection on statistics for biologists contains articles on many of the points above.

Software and code

Policy information about availability of computer code

Data collection	No software was used for data collection.
Data analysis	Statistical analyses were performed using GraphPad Prism v.10. selecting the appropriate transformations and test as indicated in the Materials and Methods. One-way ANOVA with Dunnett's multiple comparisons test was applied to compare three or more groups. An unpaired t-test was performed to compare two groups. Adjusted p values as: ns>0.05; *p≤0.05; **p≤0.01; ***p≤0.001; ****p≤0.0001

For manuscripts utilizing custom algorithms or software that are central to the research but not yet described in published literature, software must be made available to editors and reviewers. We strongly encourage code deposition in a community repository (e.g. GitHub). See the Nature Portfolio guidelines for submitting code & software for further information.

Data

Policy information about availability of data

All manuscripts must include a data availability statement. This statement should provide the following information, where applicable:

- Accession codes, unique identifiers, or web links for publicly available datasets
- A description of any restrictions on data availability
- For clinical datasets or third party data, please ensure that the statement adheres to our policy

The different accession numbers (obtained from the NCBI database) for reference genomes are on Table S1 (Phages used in this study) and Table S2 (Tai immune systems in mobile genetic elements: Genomes and characteristics). The WGS data generated in this study have been deposited in the NCBI SRA database under accession code BioProject PRJNA1069670 [https://www.ncbi.nlm.nih.gov/bioproject/PRJNA1069670]. We use ColabFold v1.5.5 AlphaFold2 using MMseqs2 server to

obtain the trimeric structure of c-terminal J proteins of HK106 (residues 876-1183), phi80 (residues 913-1192) and HK97 (residues 876-1296) phages. The models have been deposited in Zenodo [https://doi.org/10.5281/zenodo.12783471].

Research involving human participants, their data, or biological material

Policy information about studies with human participants or human data. See also policy information about sex, gender (identity/presentation), and sexual orientation and race, ethnicity and racism.

Reporting on sex and gender	n/a
Reporting on race, ethnicity, or other socially relevant groupings	n/a
Population characteristics	n/a
Recruitment	n/a
Ethics oversight	n/a

Note that full information on the approval of the study protocol must also be provided in the manuscript.

Field-specific reporting

Please select the one below that is the best fit for your research. If you are not sure, read the appropriate sections before making your selection.

☒ Life sciences ☐ Behavioural & social sciences ☐ Ecological, evolutionary & environmental sciences

For a reference copy of the document with all sections, see nature.com/documents/nr-reporting-summary-flat.pdf

Life sciences study design

All studies must disclose on these points even when the disclosure is negative.

Sample size	No calculations for determining sample size were required for this study. Experiments were repeated three times with defined comparison groups, for example, wild-type against mutant; followed by statistical analysis as described.
Data exclusions	No data were excluded from the analysis.
Replication	Experiments were performed three times as independent biological replicates, which all experimental observations were reproducible for all replication attempts. Information regarding the number of independent replicates performed are included in the methods section of the manuscript, as well as in the figure legends.
Randomization	Randomization was not relevant to this study as phenotypes of strains with clearly defined genetic differences (phage content or gene mutations) were compared.
Blinding	Blinding was not relevant to this study as phenotypes of strains with clearly defined genetic differences (phage content or gene mutations) were compared.

Reporting for specific materials, systems and methods

We require information from authors about some types of materials, experimental systems and methods used in many studies. Here, indicate whether each material, system or method listed is relevant to your study. If you are not sure if a list item applies to your research, read the appropriate section before selecting a response.

Materials & experimental systems

n/a	Involved in the study	
☐	☒	Antibodies
☒	☐	Eukaryotic cell lines
☒	☐	Palaeontology and archaeology
☒	☐	Animals and other organisms
☒	☐	Clinical data
☒	☐	Dual use research of concern
☒	☐	Plants

Methods

n/a	Involved in the study	
☒	☐	ChIP-seq
☒	☐	Flow cytometry
☒	☐	MRI-based neuroimaging

Antibodies

Antibodies used	anti-flag (Sigma-Aldrich; A8592; clone M2, purified immunoglobulin, buffered aqueous glycerol solution; LOT# SLCF0816)
Validation	ELISA Titer > 20000<br>Dot Blot Limit of Dection 8 ng Minimum detection of FLAG-Fusion protein (N-BAP) when using 1:100 dilution of conjugate<br>Dot Blot Limit of Dection 32 ng Minimum detection of FLAG-Fusion protein (N-BAP) when using 1:1000 dilution of conjugate<br>Immunocytochemistry Minimum Dilution 1:100 Minimum titer of 1:100 on FLAG transfected COS cells

Plants

Seed stocks	n/a
Novel plant genotypes	n/a
Authentication	n/a
